# Predictors of Participation in a Perinatal Text Message Screening Protocol for Maternal Depression and Anxiety: Prospective Cohort Study

**DOI:** 10.2196/53786

**Published:** 2024-10-03

**Authors:** Julia Barnwell, Cindy Hénault Robert, Tuong-Vi Nguyen, Kelsey P Davis, Chloé Gratton, Guillaume Elgbeili, Hung Pham, Michael J Meaney, Tina C Montreuil, Kieran J O'Donnell

**Affiliations:** 1 Department of Human Genetics McGill University Montreal, QC Canada; 2 Research Institute of the McGill University Health Centre Montreal, QC Canada; 3 Department of Psychiatry McGill University Montreal, QC Canada; 4 Lady Davis Institute for Medical Research Jewish General Hospital Montreal, QC Canada; 5 Reproductive Psychiatry Program Departments of Psychiatry and Obstetrics and Gynecology McGill University Health Centre Montreal, QC Canada; 6 Douglas Research Centre Montreal, QC Canada; 7 Yale Child Study Center Yale School of Medicine New Haven, CT United States; 8 Department of Obstetrics, Gynecology and Reproductive Sciences Yale School of Medicine New Haven, CT United States; 9 Translational Neuroscience Program Singapore Institute for Clinical Sciences Singapore Singapore; 10 Department of Paediatrics Yong Loo Lin School of Medicine National University of Singapore Singapore Singapore; 11 Brain - Body Initiative Agency for Science, Technology & Research (A*STAR) Singapore Singapore; 12 Department of Educational and Counselling Psychology McGill University Montreal, QC Canada; 13 Department of Pediatrics McGill University Montreal, QC Canada

**Keywords:** perinatal mental health, digital screening, maternal depression, maternal anxiety, text messaging, mHealth, mobile health, pregnancy, mobile phone

## Abstract

**Background:**

Universal screening for depression and anxiety in pregnancy has been recommended by several leading medical organizations, but the implementation of such screening protocols may overburden health care systems lacking relevant resources. Text message screening may provide a low-cost, accessible alternative to in-person screening assessments. However, it is critical to understand who is likely to participate in text message–based screening protocols before such approaches can be implemented at the population level.

**Objective:**

This study aimed to examine sources of selection bias in a texting–based screening protocol that assessed symptoms of depression and anxiety across pregnancy and into the postpartum period.

**Methods:**

Participants from the Montreal Antenatal Well-Being Study (n=1130) provided detailed sociodemographic information and completed questionnaires assessing symptoms of depression (Edinburgh Postnatal Depression Scale [EPDS]) and anxiety (State component of the State-Trait Anxiety Inventory [STAI-S]) at baseline between 8 and 20 weeks of gestation (mean 14.5, SD 3.8 weeks of gestation). Brief screening questionnaires, more suitable for delivery via text message, assessing depression (Whooley Questions) and anxiety symptoms (Generalized Anxiety Disorder 2-Item questionnaire) were also collected at baseline and then via text message at 14-day intervals. Two-tailed *t* tests and Fisher tests were used to identify maternal characteristics that differed between participants who responded to the text message screening questions and those who did not. Hurdle regression models were used to test if individuals with a greater burden of depression and anxiety at baseline responded to fewer text messages across the study period.

**Results:**

Participants who responded to the text messages (n=933) were more likely than nonrespondents (n=114) to self-identify as White (587/907, 64.7% vs 39/96, 40.6%; *P*<.001), report higher educational attainment (postgraduate: 268/909, 29.5% vs 15/94, 16%; *P*=.005), and report higher income levels (CAD $150,000 [a currency exchange rate of CAD $1=US $0.76 is applicable] or more: 176/832, 21.2% vs 10/84, 11.9%; *P*<.001). There were no significant differences in symptoms of depression and anxiety between the 2 groups at baseline or postpartum. However, baseline depression (EPDS) or anxiety (STAI-S) symptoms did predict the total number of text message time points answered by participants, corresponding to a decrease of 1% (e^β^=0.99; *P*<.001) and 0.3% (e^β^=0.997; *P*<.001) in the number of text message time points answered per point increase in EPDS or STAI-S score, respectively.

**Conclusions:**

Findings from this study highlight the feasibility of text message–based screening protocols with high participation rates. However, our findings also highlight how screening and service delivery via digital technology could exacerbate disparities in mental health between certain patient groups.

## Introduction

### Perinatal Mental Health

Perinatal mood and anxiety disorders such as depression and anxiety are among the most common complications of pregnancy affecting as many as 20% of pregnant and postpartum individuals [1]. Failure to identify those at risk of adverse perinatal mental health outcomes can have negative consequences for both mother and child [2]. Maternal suicide is a leading cause of maternal death in high-income countries [3-5], while maternal prenatal depression and anxiety associate with an increased risk of preterm birth and low birth weight [6-9], child socioemotional and behavioral difficulties [10-17], and clinically significant psychiatric symptoms in adolescence and early adulthood [15,18-21]. Cost analyses from the United States, the United Kingdom, Australia, and Canada highlight the significant economic impact of untreated perinatal mood and anxiety disorders [22-25]. As such, the early detection and the appropriate treatment of maternal depression and anxiety are public health priorities [5].

Given the high prevalence and adverse consequences of perinatal mood and anxiety disorders, several countries now recommend universal screening for maternal depression and anxiety using validated questionnaires beginning in pregnancy [26-30]. In contrast, the Canadian Task Force on Preventive Health Care recently recommended against questionnaire-based screening [31], in part due to the time-consuming nature of these assessments. The use of brief screening instruments and remote screening approaches delivered using personal mobile devices may help overcome barriers to the implementation of universal screening for maternal perinatal depression and anxiety [32-34].

### Mobile Health Perinatal Mental Health Screening

In Canada, approximately 96% of individuals aged 15 to 44 years own a smartphone [35], with comparable smartphone ownership rates in other countries including the United States [36]. The widespread availability of smartphone devices has led to increased interest in the use of personal mobile devices to deliver health care and public health services, termed “mobile health” (mHealth) [37,38]. mHealth encompasses a variety of approaches to identify, treat, or prevent adverse health outcomes including mental illness [39-46].

The most common mHealth approach is text messaging, which has been used for communication (eg, providing appointment reminders and improving patient adherence with treatment), intervention (eg, monitoring chronic conditions and providing psychological support), and patient data collection (eg, self-reported questionnaires screening for symptom levels) [39,47-51]. Text messaging allows for more timely and repeated self-report symptom capture with minimal burden [47]. A growing number of studies have used text messaging as a tool to collect self-report patient data in the perinatal period (pregnancy through 1 year postpartum) [40,52,53]. Studies in Canada and the United States have found text message–based mental health screening to be acceptable and feasible when compared to paper-based screening during the perinatal period [52-54], with increased participant satisfaction reflecting an increased perception of privacy and anonymity [52,54]. Few studies to date have examined potential selection biases (ie, sociodemographic and mental health factors) that may influence participation in a text message–based screening protocols [52]. In the United States, lower levels of participant engagement in digital health interventions were observed in racialized groups including Black and Latino communities [55]. To date, there are no Canadian reports on patient engagement in text message–based perinatal mental health screening particularly among racialized or low-income individuals [52,56-60].

In this study, we sought to identify factors that predict participation in a text message–based mental health screening protocol within a diverse, longitudinal cohort in Canada as a first step toward assessing the feasibility of using mHealth approaches to screen maternal mental health at the population level.

## Methods

### Ethical Considerations

Informed consent was obtained from all study participants, and the option to opt out of the study was provided to all participants. Ethics approval for the study was granted by Saint Mary’s Hospital Research Ethics Board (SM-18-27, MP-18-20190500) in accordance with the Helsinki Declaration of 1975. Participants selected their preferred language (French or English) during enrollment with all subsequent data collected in their language of choice. Participants were compensated for completing self-report questionnaires at recruitment, in mid-late pregnancy, and 2 postpartum time points with a CAD $10 (a currency exchange rate of CAD $1=US $0.76 is applicable) e-gift card per time point.

### Recruitment

The Montreal Antenatal Well-Being Study (MAWS) is a cohort of 1130 pregnant participants recruited between August 2019 and March 2021. Participants were recruited from prenatal care clinics associated with 3 major birth centers in Montreal, Quebec (Saint-Mary’s Hospital, Sainte-Justine Mother and Child University Hospital Center, and Lasalle Hospital). Following the onset of the COVID-19 pandemic, participants were also recruited through self-selection using targeted advertising on social media (Facebook). Eligibility criteria included being between 8 and 20 weeks of gestation; reading proficiency in French or English; aged 18 years or older; and owning a smartphone, tablet, or personal computer.

### Measures

#### Sociodemographic Information

**Figure 1:**
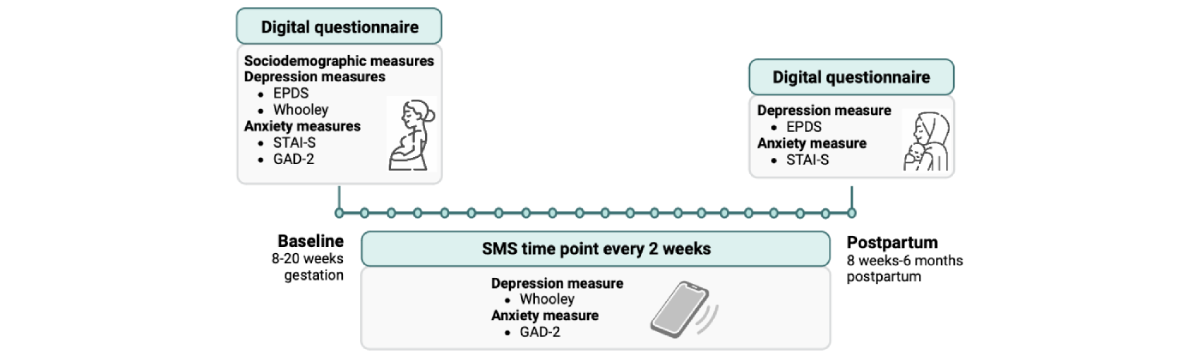
Data collection timeline of sociodemographic and mental health measures in the Montreal Antenatal Well-Being Study. EPDS: Edinburgh Postnatal Depression Scale; GAD-2: Generalized Anxiety Disorder 2-Item; STAI-S: State component of the State-Trait Anxiety Inventory.

Sociodemographic data, including maternal age, race and ethnicity, immigration status, education level, income level, and history of mental health diagnosis were collected via self-report using a secure digital platform for data capture (REDCap [Research Electronic Data Capture]; Vanderbilt University) at baseline between 8 and 20 (mean 14.50, SD 3.80) weeks of gestation (Figure 1).

#### Maternal Mental Health

Maternal depression and anxiety symptoms were assessed at baseline (recruitment) and at approximately 8 weeks postpartum (mean 8.73, SD 3.73 weeks) using validated clinical instruments through REDCap. Participants received a unique link via email at baseline and postpartum to complete their digital questionnaires on their own smartphones, tablets, or personal computers (Figure 1).

Maternal symptoms of depression were assessed using the Edinburgh Postnatal Depression Scale (EPDS). The EPDS is a widely used and validated 10-item self-report depression screening tool (Table S1 in Multimedia Appendix 1) with scores ranging from 0 to 30. EPDS sensitivity and specificity estimates range from 38% to 43% and 98% to 99%, respectively [61]. Maternal symptoms of anxiety were assessed using the State component of the State-Trait Anxiety Inventory (STAI-S). The STAI-S scale is a 20-item, self-report scale commonly used to measure an individual’s anxiety symptoms at the time of assessment (Table S1 in Multimedia Appendix 1). Each item is measured on a 4-point Likert scale. Higher scores indicate greater state anxiety symptoms. Internal consistency coefficients for the scale have ranged from 0.86 to 0.95; test-retest reliability coefficients have ranged from 0.65 to 0.75 [62]. Depression and anxiety symptoms of clinical concern are defined as scores 13 on the EPDS and 40 on the STAI-S, respectively (Table S1 in Multimedia Appendix 1).

### Text Message–Based Screening Protocol

**Figure 2:**
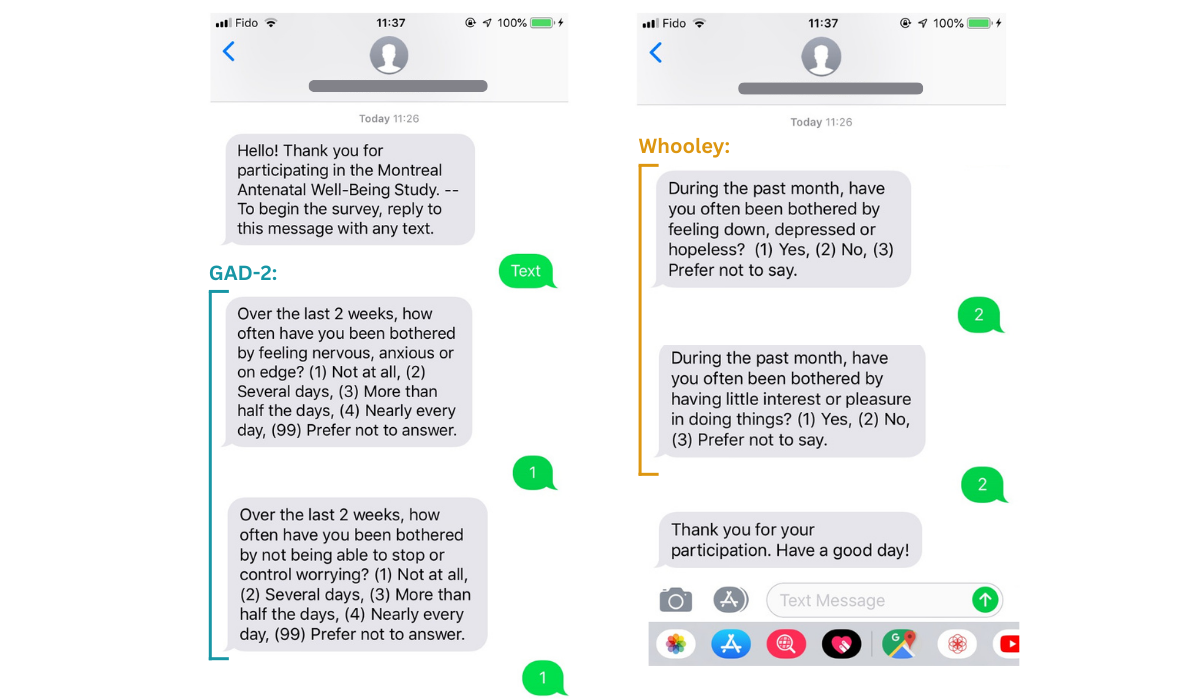
Text messages sent to Montreal Antenatal Well-Being Study participants’ personal smartphones at 14-day intervals from enrollment in the study until 8 weeks postpartum. GAD-2: Generalized Anxiety Disorder 2-Item.

Text message–based screening was performed using REDCap-Twilio integration (Figure 2 and Table S2 in Multimedia Appendix 1). Participants received their first text message time point 14 days after enrollment and then at 14-day intervals until the participant reached 8 weeks postpartum. If the survey was not initiated after the first text message, participants were prompted with a reminder text message sent 24 hours and then 48 hours after the initial text message. If participants failed to respond to 5 consecutive text message time points, no further messages were sent. A text message time point screening assessment consisted of 4 questions sent via separate text messages, which assessed symptoms of depression (Whooley Questions) [63] and anxiety (Generalized Anxiety Disorder 2-Item [GAD-2] questionnaire) [64], using 2 questions for each construct.

The Whooley Questions probe symptoms of depressed mood and anhedonia (Table S1 in Multimedia Appendix 1) and are used for routine screening of maternal depression in many countries including the United Kingdom [27]. Participants who respond “yes” to at least 1 of the 2 questions (score 1) may benefit from further evaluation (~95% sensitivity) [63]. Conversely, a negative screen suggests that no further evaluation is required. The Whooley Questions have a higher sensitivity compared to similar brief screening questionnaires for depression symptoms such as the 2-item Patient Health Questionnaire [65,66]. The GAD-2 assesses symptoms of anxiety and worry (Table S1 in Multimedia Appendix 1). Responses are scored using a Likert scale ranging from 0 to 3. An instrument score 3 has an 86% sensitivity and 83% specificity for identifying possible cases of generalized anxiety disorder and may warrant further evaluation by a clinician [64]. These 2 brief questionnaires were selected based on their suitability for delivery via text message and based on their existing use as part of universal perinatal mental health screening in the United Kingdom [27].

For this analysis, we included all maternal participants in the MAWS cohort who had received at least 1 text message time point, that is, one set of both depression and anxiety screening questions via text message. We excluded those who had withdrawn from the study prior to 8 weeks postpartum (n=60) and MAWS participants who did not receive a single text message because their area code was not covered by our service provider (n=23). This gave rise to a sample size of 1047, including 933 (89.1%) participants who responded to at least 1 text message time point and 114 (10.9%) participants who did not respond to any text message time points.

### Statistical Analyses

We defined “respondents” as participants who responded to at least 1 text message time point (n=933). “Nonrespondents” were defined as participants who did not respond to any text message time points (n=114). Fisher tests with Monte-Carlo simulations (categorical variables) and 2-tailed *t* tests (continuous variables) were used to investigate if any sociodemographic or mental health measures collected at baseline and postpartum significantly differed between respondents and nonrespondents.

In addition, hurdle regression models using a log link function were used to determine associations between participants’ total number of text message time points completed and participants’ sociodemographic variables or mental health, adjusting for the number of text message time points each participant received. Hurdle models considered the number of text message time points completed by participants according to its two possible outcomes: (1) zero time points completed, the outcome of nonrespondents (zero model); and (2) a positive number of time points completed, the outcome of respondents (count model) [67,68]. Binomial logistic regression with a log link was used for the nonrespondent zero model. On the other side of the “hurdle,” a zero-truncated Poisson distribution was used for the respondent count model. For categorical sociodemographic variables, the relative difference between the data fitting a null model containing no predictor variables and a model containing each of the sociodemographic variables individually was tested with a likelihood ratio test. For continuous baseline mental health measures, Exp (β), or the exponential value of the unstandardized coefficient β (e^β^), provided the incidence risk ratio for the count model, that is, the predicted ratio of the number of text message time points completed per unit increase in the predictor variables, whereas Exp (β) in the zero model provided the odds ratio. Adjusted hurdle models considered predictors of interest together with relevant covariates including self-reported race and ethnicity, income level, and education level.

Correlations (Spearman for ordinal and Pearson for continuous variables) were computed to determine the association of sociodemographic or mental health variables collected at baseline with participants’ “response rate,” which was defined as the number of text message time points they responded to divided by the number of text message time points they received. Thus, analyses of participant response rate account for variation in gestational age at recruitment, which determined the total number of possible text message screening time points.

Finally, linear regression models were used to determine if measures of anxiety or depression from brief text message screening assessments helped to better predict postpartum depression and anxiety symptoms than assessments of mental health at baseline. Participant response rate was also considered in these models to test whether participant compliance was a better predictor of mental health outcomes than symptom data. The Akaike Information Criterion was used as a measure of model fit to determine which of a set of predictors (ie, response rate, baseline mental health scores, or scores from the brief text message–based mental health screening questions) were the best predictors of elevated scores on validated clinical measures of postpartum depression and anxiety (EPDS and STAI-S). All analyses were run using R statistical software (version 4.2.2; R Foundation for Statistical Computing). The *pscl* package was used for the hurdle regression analyses.

## Results

### Overview

Over the course of the study, participants received an average of 14 (SD 4.66) text message screening assessments (text message time points) and responded to approximately 11 (SD 6.31) of these assessments. Some participants (n=40) received only the first 2 text message time points due to a REDCap configuration error. These participants were retained, and analyses account for the total number of text message time points received. Tables S3 and S4 in Multimedia Appendix 1 present the number of text message time points sent and answered by participants at each time point, and Table 1 shows the participant text message response rate per time point. Summary statistics of the text message time points sent and answered at each gestational or postpartum week (based on reported week of gestation at study entry) are included in Table S5 in Multimedia Appendix 1.

**Table 1:** Montreal Antenatal Well-Being Study participants’ response rate per text message time point.

Time point	Response rate
1	0.78
2	0.8
3	0.77
4	0.76
5	0.74
6	0.8
7	0.81
8	0.81
9	0.82
10	0.82
11	0.84
12	0.83
13	0.82
14	0.79
15	0.76
16	0.74
17	0.74
18	0.79
19	0.65
20	0.76
21	0.8
22	0.6
23	0.6

### Participation in a Text Message–Based Screening Protocol

#### Sociodemographic Characteristics of Respondents and Nonrespondents

Participants who responded to at least 1 text message screening assessment (ie, respondents) differed from nonrespondents on several sociodemographic factors (Table 2). Fisher tests indicated that, compared to the nonrespondent group, the respondent group was comprised of more individuals who identified as White (587/907, 64.7% vs 39/96, 40.6%; *P*<.001), who were Canadian citizens (603/768, 78.5% vs 41/77, 53.2%; *P*<.001), and who spoke 2 (483/917, 52.7% vs 46/98, 46.9%; *P*=.01) or more languages (286/917, 31.2% vs 24/98, 24.5%; *P*=.01), mainly French and English (425/566, 75.1% vs 29/53, 54.7%; *P*=.004). Respondents reported higher educational attainment (postgraduate: 268/909, 29.5% vs 15/94, 16%; *P*=.005) and higher household income (CAD $150,000 or more: 176/832, 21.2% vs 10/84, 11.9%; *P*<.001) than nonrespondents (Table 2). At baseline, respondents were more likely than nonrespondents to be primiparous (never given birth: 399/827, 48.3% vs 34/83, 41%; *P*=.049; Table 2).

**Table 2:** Baseline sociodemographic characteristics of respondents and nonrespondents.

				Full sample (n=1047)		Nonrespondents (n=114, 10.9%)		Respondents (n=933, 89.1%)		Fisher test *P* value	
Maternal age—baseline (years), mean (SD)				31.92 (4.5)		31.52 (5.1)		31.96 (4.4)		.44^a^	
**Categorical characteristics**											
	**Self-reported race and ethnicity, n/n (%)**										*<.001* ^b^
		Arab and West Asian	55/1003 (5.5)		12/96 (12.5)		43/907 (4.7)				
		Black	77/1003 (7.7)		18/96 (18.8)		59/907 (6.5)				
		East Asian	36/1003 (3.6)		5/96 (5.2)		31/907 (3.4)				
		Filipino	41/1003 (4.1)		6/96 (6.3)		35/907 (3.9)				
		Latin American	37/1003 (3.7)		2/96 (2.1)		35/907 (3.9)				
		South Asian	34/1003 (3.4)		5/96 (5.2)		29/907 (3.2)				
		Southeast Asian	13/1003 (1.3)		2/96 (2.1)		11/907 (1.2)				
		White	626/1003 (62.4)		39/96 (40.6)		587/907 (64.7)				
		Other	22/1003 (2.2)		2/96 (2.1)		20/907 (2.2)				
		Mixed	62/1003 (6.2)		5/96 (5.2)		57/907 (6.3)				
	**Immigration status, n (%)**										*<.001*
		Temporary resident	54/845 (6.4)		6/77 (7.8)		48/768 (6.3)				
		Permanent resident	147/845 (17.4)		30/77 (39)		117/768 (15.2)				
		Canadian citizen	644/845 (76.2)		41/77 (53.2)		603/768 (78.5)				
	**Number of languages spoken, n (%)**										*.01*
		1	176/1015 (17.3)		28/98 (28.6)		148/917 (16.1)				
		2	529/1015 (52.1)		46/98 (46.9)		483/917 (52.7)				
		3+	310/1015 (30.5)		24/98 (24.5)		286/917 (31.2)				
	**Spoken language (French vs English), n (%)**										*.004*
		French only	113/619 (18.3)		15/53 (28.3)		98/566 (17.3)				
		English only	52/619 (8.4)		9/53 (17)		43/566 (7.6)				
		French and English bilingual	454 (73.3)		29 (54.7)		425 (75.1)				
	**Education level, n (%)**										*.005*
		Secondary 5 or lower	80/1003 (8)		14/94 (14.9)		66/909 (7.3)				
		Prebachelors	258/1003 (25.7)		30/94 (31.9)		228/909 (25.1)				
		Bachelors	382/1003 (38.1)		35/94 (37.2)		347/909 (38.2)				
		Postgraduate	283/1003 (28.2)		15/94 (16)		268/909 (29.5)				
	**Household income (CAD $)^c^, n (%)**										*<.001*
		Less than 34,999	94/916 (10.3)		21/84 (25)		73/832 (8.8)				
		35,000 to 49,999	67/916 (7.3)		10/84 (11.9)		57/832 (6.9)				
		50,000 to 74,999	149/916 (16.3)		15/84 (17.9)		134/832 (16.1)				
		75,000 to 99,999	164/916 (17.9)		12/84 (14.3)		152/832 (18.3)				
		100,000 to 149,999	256/916 (28)		16/84 (19)		240/832 (28.8)				
		150,000 or more	186/916 (20.3)		10/84 (11.9)		176/832 (21.2)				
	**Relationship status, n (%)**										.07
		In couple	947/972 (97.4)		85/90 (94.4)		862/882 (97.7)				
		Single	25/972 (2.6)		5/90 (5.6)		20/882 (2.3)				
	**Number of previous births, n (%)**										*.05*
		Never given birth	433/910 (47.6)		34/83 (41)		399/827 (48.3)				
		1	316/910 (34.7)		25/83 (30.1)		291/827 (35.2)				
		2	108/910 (11.9)		16/83 (19.3)		92/827 (11.1)				
		3+	53/910 (5.8)		8/83 (9.6)		45/827 (5.4)				
	**Mental health diagnoses, n (%)**										.15
		Yes	225/1007 (22.3)		15/94 (16)		210/913 (23)				
		No	782/1007 (77.7)		79/94 (84)		703/913 (77)				

^a^Two-tailed *t* test *P* value.

^b^Values in italics format indicate statistical significance.

^c^A currency exchange rate of CAD $1=US $0.76 is applicable.

#### Baseline and Postpartum Mental Health of Respondents and Nonrespondents

Respondents and nonrespondents reported similar levels of depression and anxiety symptoms both at baseline and postpartum (Table 3; see also Table S6 in Multimedia Appendix 1). The number of individuals who reported having received a mental health diagnosis was not significantly different between groups (*P*=.15; Table 2).

**Table 3:** Mental health measure scores of respondents and nonrespondents.

Continuous measures		Full sample (n=1047)			Nonrespondents (n=114, 10.9%)			Respondents (n=933, 89.1%)			*P* value^a^
		Participants, n	Mean (SD)	Participants, n		Mean (SD)	Participants, n		Mean (SD)		
**Baseline**											
	EPDS^b^ score	997	6.5 (4.79)	91		6.98 (4.55)	906		6.45 (4.82)	.30	
	STAI-S^c^ score	985	33.97 (11.28)	88		35.67 (12.05)	897		33.81 (11.2)	.17	
**Postpartum**											
	EPDS score	854	6.07 (4.8)	54		5.74 (3.86)	800		6.09 (4.86)	.53	
	STAI-S score	827	32.48 (11.1)	46		30.96 (8.68)	781		32.57 (11.22)	.23	

^a^Two-tailed *t* tests.

^b^EPDS: Edinburgh Postnatal Depression Scale (scores were prorated if ≥80% data available).

^c^STAI-S: State component of the State-Trait Anxiety Inventory (scores were prorated if ≥80% data available).

### Predictors of the Number of Text Message Screening Time Points Completed

Next, we asked which factors predicted the total number of responses to regular (every 2 weeks) text message screening assessments across pregnancy (see Table S7 in Multimedia Appendix 1 for bivariate correlations). Unadjusted bivariate hurdle models indicated that there were significant differences in the number of text message time points completed based on self-reported race and ethnicity (*P*<.001), immigration status (*P*<.001), number of languages spoken fluently (*P*=.002), French-English bilingualism (*P*<.001), education level (*P*<.001), household income level (*P*<.001), relationship status (*P*<.001), and number of previous births (*P*=.01; Table 4), with self-reported race and ethnicity having the strongest effect. Table 4 also describes the results from the adjusted hurdle model where maternal race and ethnicity (*P*<.001), education (*P*=.001), household income (*P*=.02), and relationship status (*P*=.03) remained significantly and independently associated with the total number of text message time points completed by participants. Maternal age at baseline was not significantly associated with the likelihood of responding to one (or more) text message time points after considering relevant covariates (Table S8 in Multimedia Appendix 1).

Measures of maternal mental health at baseline also predicted the total number of text message time points completed across the duration of the study (Table 5 and Table S9 in Multimedia Appendix 1). A 1-point increase in EPDS and STAI-S scores at baseline was associated with a respective decrease of 1% (e^β^=0.99) and 0.3% (e^β^=0.997) in the average number of text message time points answered by participants (Table 5). Thus, for each SD increase in maternal depression or anxiety scores, the average number of text message time points completed was 4.7% and 3.3% lower, respectively. In hurdle models adjusted for self-reported race and ethnicity, education level, and household income level, baseline EPDS (*P*<.001) and STAI-S scores (*P*=.03) remained significant predictors of the total number of text message-based screening assessments completed (Table 5). In contrast, a previous mental health diagnosis (reported at baseline) did not improve the prediction of number of text message time points completed by participants in both the unadjusted (*P*=.17) and adjusted (*P*=.91) models (Table 4).

**Table 4:** Associations between baseline categorical sociodemographic variables and participants’ number of text message time points completed.

Categorical variables		Participants, n	Log-likelihood null	Log-likelihood variable	*P* value
**Unadjusted**					
	Self-reported ethnicity	1003	–2791.06	–2738.32	*<.001* ^a^
	Immigration status	845	–2343.97	–2321.83	*<.001*
	Number of languages spoken	1015	–2841.3	–2832.65	*.002*
	Spoken language (French vs English)	619	–1703	–1691.03	*<.001*
	Education level	1003	–2804.16	–2778.46	*<.001*
	Household income	916	–2519.26	–2486.46	*<.001*
	Relationship status	972	–2720.14	–2710.38	*<.001*
	Number of previous births	910	–2546.73	–2538.61	*.01*
	Mental health diagnosis	1007	–2817.01	–2815.23	.17
**Adjusted^b^**					
	Self-reported ethnicity	905	–2443.98	–2417.47	*<.001*
	Immigration status	752	–1993.68	–1989.02	.05
	Number of languages spoken	905	–2417.47	–2416.59	.78
	Spoken language (French vs English)	573	–1510.95	–1509.17	.47
	Education level	905	–2428.91	–2417.47	*.001*
	Household income	905	–2427.83	–2417.47	*.02*
	Relationship status	870	–2322.42	–2319.03	*.03*
	Number of previous births	821	–2189.75	–2186.93	.46
	Mental health diagnosed	903	–2412.25	–2412.16	.91

^a^Values in italics format indicate statistical significance.

^b^Adjusted log-likelihood null models (with categorical variables) include self-reported race and ethnicity, income level, and education level as covariates.

**Table 5:** Associations between baseline mental health measure scores and participants’ number of text message time points completed.

Continuous variables		n	β Count	SE β count	Exp (β) count^a^	*P* value count	β Zero	SE β zero	Exp (β) zero^b^	*P* value zero
**Unadjusted**										
	EPDS^c^—baseline	997	–0.01	0.002	0.99	*<.001* ^d^	–0.02	0.02	0.979	.37
	STAI-S^e^—baseline	985	–0.003	0.001	0.997	*<.001*	–0.01	0.01	0.988	.23
**Adjusted^f^**										
	EPDS—baseline	893	–0.008	0.002	0.992	*<.001*	0.01	0.03	1.013	.62
	STAI-S—baseline	885	–0.002	0.001	0.998	*.03*	–0.004	0.01	0.996	.69

^a^Exp (β), or the exponential value of β (e^β^), provides the incidence risk ratio for the count model, that is, the association between baseline mental health measure scores and the incidence rate of participants responding to a positive number of text message time points.

^b^Exp (β) provides the odds ratio in the zero model, that is, the association between baseline mental health measure scores and the odds of responding to at least 1 text message time point.

^c^EPDS: Edinburgh Postnatal Depression Scale.

^d^Values in italics format indicate statistical significance.

^e^STAI-S: State component of the State-Trait Anxiety Inventory.

^f^Adjusted hurdle regression models include ethnicity, income level, and education level as covariates.

### Text Message Response Rate Does Not Outperform Brief Symptom Measures in the Prediction of Postpartum Anxiety and Depression Symptoms

Given the associations we observed between baseline measures of maternal mental health and the number of text message time points participants responded to, we next asked if participant text message response rate was a better predictor of postpartum mental health than symptom data from brief screening tools. Specifically, these models tested if text message response rate was a better predictor of depression and anxiety symptoms (measured by EPDS and STAI-S) at approximately 8 weeks postpartum than scores derived from the Whooley and GAD-2 questionnaires. Text message response rate did not improve the prediction of postpartum depression or anxiety (Table S10 in Multimedia Appendix 1). Similar results were found in the adjusted models, suggesting that measures of selection bias (as reflected by text message response rate) were not a significant predictor of postpartum depression or anxiety symptoms (Table S10 in Multimedia Appendix 1). In contrast, we found that participant GAD-2 scores collected via text message proximal (average 7.3, SD 21.7 days) to the postpartum screening assessment provided the strongest prediction of maternal postpartum depression and anxiety symptom levels (Table 6).

**Table 6:** Comparison of predictors (based on model fit statistics) of postpartum depression and anxiety symptoms. Model fit was estimated using the Akaike information criterion (AIC) with lower values indicating a better fit or prediction.

Outcome	Predictor								
	Response rate	Baseline measures			Last gestation measures			Last postpartum measures	
		STAI-S^a^ score	EPDS^b^ score	GAD-2^c^ score		Whooley score	GAD-2 score		Whooley score
EPDS score—postpartum	3959.3	3832.5	3829.27	3893.5		3917.27	*3722.56* ^d^		3813.89
STAI-S score—postpartum	5063.27	4896.73	4963.46	4985.09		5002.80	*4840.15*		4920.96

^a^STAI-S: State component of the State-Trait Anxiety Inventory.

^b^EPDS: Edinburgh Postnatal Depression Scale.

^c^GAD-2: Generalized Anxiety Disorder 2-Item.

^d^The lowest AIC (best fit) for each outcome is set in italics format.

## Discussion

### Principal Findings

This study provides a comprehensive analysis of sociodemographic and mental health selection bias in participation in a text message–based perinatal mental health screening protocol. Overall, we found some evidence of selection bias that was patterned by maternal characteristics including race and ethnicity, income level, education, and parity. While we did not find strong evidence for an impact of maternal mental health on initial participation in our text message–based screening protocol, we did observe fewer total text message time points completed based on baseline maternal depression and anxiety symptoms. These findings suggest that text messaging may be a useful tool in the context of perinatal mental health screening. However, this study highlights important individual-level factors that may impact the effectiveness of text message–based mental health screening.

### Sociodemographic and Mental Health Factors Predict Participation in Text Message–Based Screening

This study identified several sociodemographic variables that were associated with initial participation in a text message–based mental health screening protocol, and several of these factors also influenced the total number of text message time points completed over time. Specifically, respondents were more likely to identify as White, report Canadian citizenship, speak more languages (predominantly French-English bilingualism), have higher educational attainment, have higher income, and have fewer children. Self-reported race and ethnicity, education level, household income level, and relationship status were also associated with the number of text message time points answered by respondents. Our findings are consistent with previous studies, which have identified higher engagement with mobile-based health interventions among socially advantaged groups, reflecting potential challenges faced among disadvantaged groups, such as time constraints, differences in communication needs and preferences, and varying levels of literacy, trust, and comfort with digital technology [69,70]. Previous studies demonstrating the feasibility of text message– or mobile-based perinatal mental health screening have generally been performed in well-educated, higher-income cohorts [52] or in smaller cohorts than this study [71]. For example, a previous Canadian study focused predominantly on women with a university degree (865/937, 92%), which contrasts with this study (665/1003, 66% college-educated). Further empirical and qualitative studies are needed to parse the role of these factors and their interplay in the prediction of participation in text message–based screening. This work should include racially, culturally, and economically diverse samples and ideally incorporate qualitative studies to better understand individual-level factors that may act as barriers to participation in a text message–based screening of perinatal mental health.

Maternal depression and anxiety symptoms as well as previous mental health diagnoses were comparable across respondents and nonrespondents at baseline and postpartum. Thus, the likelihood of participation in a text message–based mental health screening protocol does not appear to vary as a function of maternal mental health. This finding provides supportive evidence of the utility of this approach to assess mental health symptoms in pregnant individuals. However, we did observe a significant negative association between maternal symptoms of depression and anxiety at baseline and the total number of text message time points answered over time. Our finding is consistent with a UK-based study that found that a history of depression and a history of use of psychiatric medication were negatively associated with the use of a postnatal depression screening app [71]. Similarly, a Japanese perinatal cohort reported that maternal psychological distress during pregnancy correlated with nonresponsiveness to follow-up questionnaires in the postpartum period [72]. Collectively, these findings suggest that the burden of repeated mental health assessments may lead to increased attrition among vulnerable groups. Thus, the frequency of mental health screening assessments is an important consideration for public health initiatives that seek to repeatedly assess maternal mental health across the perinatal period.

### Response Rate and Brief Screening Scores as Predictors of Postpartum Depression and Anxiety Symptoms

Anxiety symptoms, as reported using the GAD-2 questionnaire sent via text message, emerged as the strongest predictor of postpartum symptoms, while participants’ text message response rate was not significantly associated with postpartum mental health symptoms. Specifically, the GAD-2 score most proximal to the postpartum assessment of depression and anxiety, with an average interval of 7.3 days between these assessments, was the best predictor of postpartum symptom levels. This finding is unsurprising as closely spaced assessments of similar constructs are likely to be highly intercorrelated. However, we did note a stronger prediction of both postpartum depression and anxiety symptoms by GAD-2 scores than scores derived from the Whooley Questions, which assess symptoms of depression. This finding is consistent with a previous report highlighting a robust association between prenatal anxiety and postpartum depression [73]. Our multivariable analyses show that the GAD-2 is a helpful brief screening tool that captures additional variance in postpartum anxiety and depression symptoms (beyond that explained by sociodemographic factors) [64,74].

Overall, the adoption of digital, mobile-based short-form perinatal mental health screening has the potential to address clinical barriers such as time and resource constraints. Consistent with previous findings, the high rate of participation from the MAWS sample in the text message screening protocol (933/1047, 89.1%) also suggests that text message–based screening may be appealing to a broad section of pregnant and postpartum individuals [52-54]. Our findings emphasize the need to identify and remove the barriers that contribute to lower patient engagement in digital screening protocols among disadvantaged groups who are at higher risk of developing a perinatal mental health disorder [75]. Such barriers could include time constraints, reduced access to a mobile device, language barriers, mistrust of health institutions, and stigma associated with mental health, among others. Overcoming these barriers may help more fully realize the clear potential of text messaging technology to reduce inequitable access to perinatal mental health care.

### Limitations

This study is not without limitations. First, this study only included participants who owned a smartphone, tablet, or personal computer, and due to the COVID-19 pandemic, 216 (20.6%) of the 1047 MAWS cohort sample participants were recruited using targeted advertisements on social media. Thus, the participation rate in our text message–based screening protocol (933/1047, 89.1%) may be higher than studies that focus on a more general perinatal population. However, we note that only 1 (0.03%) of 3761 individuals approached to participate in MAWS did not own a smartphone, tablet, or personal computer. Likewise, our participation rate was similar to Lawson et al [52] (930/937, 99%), who carried out a text message–based screening protocol in a postpartum cohort.

Second, although studies have previously demonstrated the high accuracy and internal consistency of existing clinical screening tools like those administered to MAWS participants (Whooley, GAD-2, EPDS, and STAI-S) across ethnically diverse populations [76-80], many of these instruments contain idioms that may not translate well to different languages and may lack sensitivity in the conceptualization of symptoms of perinatal maternal depression and anxiety across different cultures. Future studies would benefit from considering culturally relevant research methodologies and questionnaires for digital screening of perinatal mental health [32].

These limitations notwithstanding, a major strength of the study design is that it allowed us to collect longitudinal data on participants who did not participate in the text message component of the study. Studies whose sole focus is on testing the feasibility of a text message–based screening protocol are, by nature of their design, unable to collect longitudinal data on nonengaged participants. Our findings therefore bring much-needed insights into the sociodemographic and mental health profile of pregnant individuals who choose to participate in and consistently respond to text message–based mental health screening assessments.

### Conclusions

New approaches are required to better identify and treat perinatal mood and anxiety disorders, which cause profound human distress and result in large economic costs. Our study provides preliminary support for the feasibility and utility of a text message–based perinatal mental health screening protocol; the first evidence of this kind derived from a bilingual Canadian cohort. However, our findings also highlight how digital technologies could contribute to further disparities in mental health screening and treatment, an equity issue that should be a central focus for health policy formation.

## Appendix

Multimedia Appendix 1 Summary information and statistics on the Montreal Antenatal Well-Being Study’s text message time points.

## Abbreviations

EPDS: Edinburgh Postnatal Depression Scale
Exp (β), eβ: exponential value of β
GAD-2: Generalized Anxiety Disorder 2-Item
MAWS: Montreal Antenatal Well-Being Study
mHealth: mobile health
REDCap: Research Electronic Data Capture
STAI-S: State component of the State-Trait Anxiety Inventory
