# Supplementary material for: Predictors of Participation in a Perinatal Text Message Screening Protocol for Maternal Depression and Anxiety: Prospective Cohort Study
Source: JMIR Pediatr Parent. 2024 Oct 3;7:e53786. doi: 10.2196/53786 (PMC11487212; doi:10.2196/53786)
Supplement: Multimedia Appendix 1 [file pediatrics_v7i1e53786_app1.docx]

**Table S1.** Description of full-length and brief mental health measures administered in the MAWS cohort.

| **Measures** | | **Length of Measures** | **Clinical Cut-off Score** | **Description of Measure** |
| --- | --- | --- | --- | --- |
| **Depression** | Edinburgh Postnatal Depression Scale (EPDS) | 10 items | ≥13 | A self-report scale designed to screen for perinatal depression, with possible scores ranging from 0-30. Scores ≥13 are used to identify women with clinically concerning depression symptoms. For a cut-off of 13 on the EPDS, sensitivity ranges from 38 to 43% (depending on trimester) and specificity is 98–99% [61]. |
|  | Whooley Questions for Depression Screening | 2 items | ≥1 | A brief self-report scale with yes/no responses. A positive test (~95% sensitivity) is a response of “yes” to one or both questions and identifies patients who may benefit from further evaluation. A negative test (responding “no” to both questions) rules out the need for further evaluation. The Whooley Questions (~65% specificity) cannot be used to diagnose or measure the severity of depression [63]. |
| **Anxiety** | State-State Anxiety Inventory (STAI-S) | 20 items | ≥40 | A self-report scale, commonly used measure of state anxiety. All items are rated on a 4-point scale (e.g., from “Almost Never” to “Almost Always”). Higher scores indicate greater anxiety. Internal consistency coefficients for the scale have ranged from .86 to .95; test-retest reliability coefficients have ranged from .65 to .75 [62]. |
|  | Generalized Anxiety Disorder 2-Item (GAD-2) | 2 items | ≥3 | A brief self-report scale for initial screening of generalized anxiety disorder. A score of 3 points (86% sensitivity and 83% specificity) is the preferred cut-off for identifying possible cases of generalized anxiety disorder and in which further diagnostic evaluation is warranted [64]. |

**Table S2.** Whooley and GAD-2 questions administered in the MAWS cohort in English and French.

| **Measures** | **Questions in English** | **Questions in French** |
| --- | --- | --- |
| Whooley Questions for Depression Screening | During the past month, have you often been bothered by feeling down, depressed or hopeless?   1. Yes 2. No 3. Prefer not to answer | Au cours du dernier mois, vous êtes-vous sentie triste, déprimée ou désespérée?   1. Oui 2. Non 3. Je préfère ne pas répondre |
|  | During the past month, have you often been bothered by having little interest or pleasure in doing things?   1. Yes 2. No 3. Prefer not to answer | Au cours du dernier mois, avez-vous ressenti un manque d’intérêt ou de plaisir à réaliser des tâches de la vie quotidienne?   1. Oui 2. Non 3. Je préfère ne pas répondre |
| Generalized Anxiety Disorder 2-Item (GAD-2) | Over the last 2 weeks, how often have you been bothered by feeling nervous, anxious or on edge?   1. Not at all 2. Several days 3. More than half the days 4. Nearly every day 5. Prefer not to answer | Au cours des deux dernières semaines, à quelle fréquence vous êtes-vous sentie nerveuse, anxieuse ou les nerfs à vif?   1. Pas du tout 2. Plusieurs jours 3. Plus de la moitié des jours 4. Presque tous les jours 5. Je préfère ne pas répondre |
|  | Over the last 2 weeks, how often have you been bothered by not being able to stop or control worrying?   1. Not at all 2. Several days 3. More than half the days 4. Nearly every day 5. Prefer not to answer | Au cours des deux dernières semaines, à quelle fréquence avez-vous été incapable d’arrêter de vous inquiéter ou de contrôler vos inquiétudes?   1. Pas du tout 2. Plusieurs jours 3. Plus de la moitié des jours 4. Presque tous les jours 5. Je préfère ne pas répondre |

**Table S3.** Number of text message timepoints sent per timepoint.

| **SMS** | **All** | | | **French only** | | | **English only** | | |
| --- | --- | --- | --- | --- | --- | --- | --- | --- | --- |
|  | **Number sent** | **% of participants** | **% total** | **Number sent** | **% of participants** | **% total** | **Number sent** | **% of participants** | **% total** |
| timepoint #1 | 1047 | 100.00 | 7.15 | 659 | 100.00 | 7.09 | 388 | 100.00 | 7.26 |
| timepoint #2 | 1044 | 99.71 | 7.13 | 658 | 99.85 | 7.08 | 386 | 99.48 | 7.22 |
| timepoint #3 | 1003 | 95.80 | 6.85 | 619 | 93.93 | 6.66 | 384 | 98.97 | 7.18 |
| timepoint #4 | 1001 | 95.61 | 6.83 | 618 | 93.78 | 6.65 | 383 | 98.71 | 7.16 |
| timepoint #5 | 996 | 95.13 | 6.80 | 616 | 93.47 | 6.63 | 380 | 97.94 | 7.11 |
| timepoint #6 | 938 | 89.59 | 6.40 | 592 | 89.83 | 6.37 | 346 | 89.18 | 6.47 |
| timepoint #7 | 909 | 86.82 | 6.21 | 575 | 87.25 | 6.18 | 334 | 86.08 | 6.25 |
| timepoint #8 | 896 | 85.58 | 6.12 | 568 | 86.19 | 6.11 | 328 | 84.54 | 6.13 |
| timepoint #9 | 881 | 84.15 | 6.02 | 558 | 84.67 | 6.00 | 323 | 83.25 | 6.04 |
| timepoint #10 | 867 | 82.81 | 5.92 | 546 | 82.85 | 5.87 | 321 | 82.73 | 6.00 |
| timepoint #11 | 853 | 81.47 | 5.82 | 538 | 81.64 | 5.79 | 315 | 81.19 | 5.89 |
| timepoint #12 | 844 | 80.61 | 5.76 | 531 | 80.58 | 5.71 | 313 | 80.67 | 5.85 |
| timepoint #13 | 822 | 78.51 | 5.61 | 520 | 78.91 | 5.59 | 302 | 77.84 | 5.65 |
| timepoint #14 | 708 | 67.62 | 4.83 | 463 | 70.26 | 4.98 | 245 | 63.14 | 4.58 |
| timepoint #15 | 619 | 59.12 | 4.23 | 408 | 61.91 | 4.39 | 211 | 54.38 | 3.95 |
| timepoint #16 | 518 | 49.47 | 3.54 | 344 | 52.20 | 3.70 | 174 | 44.85 | 3.25 |
| timepoint #17 | 381 | 36.39 | 2.60 | 254 | 38.54 | 2.73 | 127 | 32.73 | 2.37 |
| timepoint #18 | 168 | 16.05 | 1.15 | 126 | 19.12 | 1.36 | 42 | 10.82 | 0.79 |
| timepoint #19 | 85 | 8.12 | 0.58 | 67 | 10.17 | 0.72 | 18 | 4.64 | 0.34 |
| timepoint #20 | 21 | 2.01 | 0.14 | 14 | 2.12 | 0.15 | 7 | 1.80 | 0.13 |
| timepoint #21 | 15 | 1.43 | 0.10 | 8 | 1.21 | 0.09 | 7 | 1.80 | 0.13 |
| timepoint #22 | 15 | 1.43 | 0.10 | 8 | 1.21 | 0.09 | 7 | 1.80 | 0.13 |
| timepoint #23 | 15 | 1.43 | 0.10 | 8 | 1.21 | 0.09 | 7 | 1.80 | 0.13 |

**Table S4.** Number of text message timepoints answered per timepoint.

| **SMS** | **All** | | | **French only** | | | **English only** | | |
| --- | --- | --- | --- | --- | --- | --- | --- | --- | --- |
|  | **Number answered** | **% of participants** | **% total** | **Number answered** | **% of participants** | **% total** | **Number answered** | **% of participants** | **% total** |
| timepoint #1 | 813 | 77.65 | 7.01 | 521 | 79.06 | 7.01 | 292 | 75.26 | 7.02 |
| timepoint #2 | 833 | 79.79 | 7.18 | 539 | 81.91 | 7.25 | 294 | 76.17 | 7.07 |
| timepoint #3 | 770 | 76.77 | 6.64 | 481 | 77.71 | 6.47 | 289 | 75.26 | 6.95 |
| timepoint #4 | 760 | 75.92 | 6.55 | 473 | 76.54 | 6.36 | 287 | 74.93 | 6.90 |
| timepoint #5 | 742 | 74.50 | 6.40 | 468 | 75.97 | 6.29 | 274 | 72.11 | 6.59 |
| timepoint #6 | 752 | 80.17 | 6.49 | 474 | 80.07 | 6.37 | 278 | 80.35 | 6.68 |
| timepoint #7 | 740 | 81.41 | 6.38 | 463 | 80.52 | 6.23 | 277 | 82.93 | 6.66 |
| timepoint #8 | 722 | 80.58 | 6.23 | 456 | 80.28 | 6.13 | 266 | 81.10 | 6.40 |
| timepoint #9 | 725 | 82.29 | 6.25 | 457 | 81.90 | 6.15 | 268 | 82.97 | 6.44 |
| timepoint #10 | 710 | 81.89 | 6.12 | 450 | 82.42 | 6.05 | 260 | 81.00 | 6.25 |
| timepoint #11 | 719 | 84.29 | 6.20 | 456 | 84.76 | 6.13 | 263 | 83.49 | 6.32 |
| timepoint #12 | 702 | 83.18 | 6.05 | 447 | 84.18 | 6.01 | 255 | 81.47 | 6.13 |
| timepoint #13 | 678 | 82.48 | 5.85 | 433 | 83.27 | 5.82 | 245 | 81.13 | 5.89 |
| timepoint #14 | 557 | 78.67 | 4.80 | 375 | 80.99 | 5.04 | 182 | 74.29 | 4.38 |
| timepoint #15 | 473 | 76.41 | 4.08 | 320 | 78.43 | 4.30 | 153 | 72.51 | 3.68 |
| timepoint #16 | 384 | 74.13 | 3.31 | 264 | 76.74 | 3.55 | 120 | 68.97 | 2.89 |
| timepoint #17 | 282 | 74.02 | 2.43 | 193 | 75.98 | 2.60 | 89 | 70.08 | 2.14 |
| timepoint #18 | 132 | 78.57 | 1.14 | 96 | 76.19 | 1.29 | 36 | 85.71 | 0.87 |
| timepoint #19 | 55 | 64.71 | 0.47 | 43 | 64.18 | 0.58 | 12 | 66.67 | 0.29 |
| timepoint #20 | 16 | 76.19 | 0.14 | 11 | 78.57 | 0.15 | 5 | 71.43 | 0.12 |
| timepoint #21 | 12 | 80.00 | 0.10 | 6 | 75.00 | 0.08 | 6 | 85.71 | 0.14 |
| timepoint #22 | 9 | 60.00 | 0.08 | 5 | 62.50 | 0.07 | 4 | 57.14 | 0.10 |
| timepoint #23 | 9 | 60.00 | 0.08 | 5 | 62.50 | 0.07 | 4 | 57.14 | 0.10 |

**Table S5.** Weeks of gestation or postpartum at which participants answered.

| **Gestational Weeks** | **Number answered** | **% of participants** | **% total** |
| --- | --- | --- | --- |
| Answered_SMS_gestweek7 | 1 | 0.10 | 0.01 |
| Answered_SMS_gestweek8 | 5 | 0.49 | 0.04 |
| Answered_SMS_gestweek9 | 16 | 1.57 | 0.14 |
| Answered_SMS_gestweek10 | 35 | 3.43 | 0.31 |
| Answered_SMS_gestweek11 | 66 | 6.46 | 0.58 |
| Answered_SMS_gestweek12 | 69 | 6.76 | 0.60 |
| Answered_SMS_gestweek13 | 145 | 14.20 | 1.26 |
| Answered_SMS_gestweek14 | 200 | 19.59 | 1.74 |
| Answered_SMS_gestweek15 | 225 | 22.04 | 1.96 |
| Answered_SMS_gestweek16 | 242 | 23.70 | 2.11 |
| Answered_SMS_gestweek17 | 262 | 25.66 | 2.28 |
| Answered_SMS_gestweek18 | 286 | 28.01 | 2.49 |
| Answered_SMS_gestweek19 | 318 | 31.15 | 2.77 |
| Answered_SMS_gestweek20 | 324 | 31.73 | 2.82 |
| Answered_SMS_gestweek21 | 335 | 32.81 | 2.92 |
| Answered_SMS_gestweek22 | 390 | 38.20 | 3.40 |
| Answered_SMS_gestweek23 | 365 | 35.75 | 3.18 |
| Answered_SMS_gestweek24 | 392 | 38.39 | 3.42 |
| Answered_SMS_gestweek25 | 356 | 34.87 | 3.10 |
| Answered_SMS_gestweek26 | 381 | 37.32 | 3.32 |
| Answered_SMS_gestweek27 | 358 | 35.06 | 3.12 |
| Answered_SMS_gestweek28 | 377 | 36.92 | 3.29 |
| Answered_SMS_gestweek29 | 350 | 34.28 | 3.05 |
| Answered_SMS_gestweek30 | 374 | 36.63 | 3.26 |
| Answered_SMS_gestweek31 | 349 | 34.18 | 3.04 |
| Answered_SMS_gestweek32 | 361 | 35.36 | 3.15 |
| Answered_SMS_gestweek33 | 346 | 33.89 | 3.02 |
| Answered_SMS_gestweek34 | 384 | 37.61 | 3.35 |
| Answered_SMS_gestweek35 | 335 | 32.81 | 2.92 |
| Answered_SMS_gestweek36 | 346 | 33.89 | 3.02 |
| Answered_SMS_gestweek37 | 303 | 29.68 | 2.64 |
| Answered_SMS_gestweek38 | 269 | 26.35 | 2.35 |
| Answered_SMS_gestweek39 | 146 | 14.30 | 1.27 |
| Answered_SMS_gestweek40 | 71 | 6.95 | 0.62 |
| Answered_SMS_gestweek41 | 16 | 1.57 | 0.14 |
| Answered_SMS_gestweek42 | 12 | 1.18 | 0.10 |
| Answered_SMS_gestweek43 | 6 | 0.59 | 0.05 |
| Answered_SMS_gestweek44 | 7 | 0.69 | 0.06 |
| Answered_SMS_gestweek45 | 8 | 0.78 | 0.07 |
| Answered_SMS_gestweek46 | 7 | 0.69 | 0.06 |
| Answered_SMS_gestweek47 | 4 | 0.39 | 0.03 |
| Answered_SMS_postpartumweek0 | 306 | 29.97 | 2.67 |
| Answered_SMS_postpartumweek1 | 339 | 33.20 | 2.96 |
| Answered_SMS_postpartumweek2 | 307 | 30.07 | 2.68 |
| Answered_SMS_postpartumweek3 | 325 | 31.83 | 2.83 |
| Answered_SMS_postpartumweek4 | 291 | 28.50 | 2.54 |
| Answered_SMS_postpartumweek5 | 316 | 30.95 | 2.76 |
| Answered_SMS_postpartumweek6 | 249 | 24.39 | 2.17 |
| Answered_SMS_postpartumweek7 | 192 | 18.81 | 1.67 |
| Answered_SMS_postpartumweek8 | 120 | 11.75 | 1.05 |
| Answered_SMS_postpartumweek9 | 60 | 5.88 | 0.52 |
| Answered_SMS_postpartumweek10 | 26 | 2.55 | 0.23 |
| Answered_SMS_postpartumweek11 | 18 | 1.76 | 0.16 |
| Answered_SMS_postpartumweek12 | 12 | 1.18 | 0.10 |
| Answered_SMS_postpartumweek13 | 8 | 0.78 | 0.07 |
| Answered_SMS_postpartumweek14 | 11 | 1.08 | 0.10 |
| Answered_SMS_postpartumweek15 | 7 | 0.69 | 0.06 |
| Answered_SMS_postpartumweek16 | 9 | 0.88 | 0.08 |
| Answered_SMS_postpartumweek17 | 8 | 0.78 | 0.07 |
| Answered_SMS_postpartumweek18 | 6 | 0.59 | 0.05 |
| Answered_SMS_postpartumweek19 | 4 | 0.39 | 0.03 |
| Answered_SMS_postpartumweek20 | 6 | 0.59 | 0.05 |
| Answered_SMS_postpartumweek21 | 2 | 0.20 | 0.02 |
| Answered_SMS_postpartumweek22 | 2 | 0.20 | 0.02 |
| Answered_SMS_postpartumweek23 | 2 | 0.20 | 0.02 |
| Answered_SMS_postpartumweek24 | 1 | 0.10 | 0.01 |
| Answered_SMS_postpartumweek25 | 0 | 0.00 | 0.00 |
| Answered_SMS_postpartumweek26 | 1 | 0.10 | 0.01 |

**Table S6.** Baseline mental health brief measure scores of respondents and non-respondents.

|  | **Full sample**  (n=1047) | | **Non-respondents**  (n=114, 10.9%) | | **Respondents**  (n=933, 89.1%) | |  |
| --- | --- | --- | --- | --- | --- | --- | --- |
| **Continuous Measures** | n | Mean (SD) | n | Mean (SD) | n | Mean (SD) | *P* value^a^ |
| Whooley Score - Baseline | 1001 | 0.94 (0.86) | 94 | 1.06 (0.84) | 907 | 0.93 (0.86) | .15 |
| GAD-2 Score - Baseline | 1001 | 1.49 (1.55) | 95 | 1.16 (1.21) | 906 | 1.52 (1.58) | *.008* |

^a^ from two-tailed *t* tests with Italicized values indicating statistical significance.

**Table S7.** Spearman and Pearson correlations between text message response rate and sociodemographic variables and mental health scores at baseline.

| Variable | r | *P* value ^a^ |
| --- | --- | --- |
| Categorical (Spearman) | | |
| Number of languages spoken | 0.007 | .83 |
| Education level | 0.176 | *<.001* |
| Household income | 0.162 | *<.001* |
| Relationship status | -0.075 | *.03* |
| Number of previous births | -0.113 | *.001* |
| Mental health diagnosed | -0.049 | .14 |
| Continuous (Pearson) | | |
| Maternal Age – Baseline | 0.071 | *.03* |
| Whooley – Baseline | -0.089 | *.008* |
| GAD-2 – Baseline | -0.062 | .06 |
| EPDS – Baseline | -0.166 | *<.001* |
| STAI-S – Baseline | -0.119 | *<.001* |

^a^ Italicized values indicate statistical significance.

**Table S8.** Associations between baseline maternal age and participants’ number of text message timepoints completed.

| **Continuous Variables** | n | β Count | SE β Count | Exp (β) Count ^a^ | *P* value Count | β Zero | SE β Zero | Exp (β) Zero ^b^ | *P* value Zero |
| --- | --- | --- | --- | --- | --- | --- | --- | --- | --- |
| Unadjusted | | | | | | | | | |
| Maternal Age – Baseline (years) | 985 | 0.004 | 0.002 | 1.004 | .08 | 0.02 | 0.03 | 1.02 | .44 |
| Adjusted ^c^ | | | | | | | | | |
| Maternal Age – Baseline (years) | 871 | 0.001 | 0.002 | 1.001 | .73 | -0.03 | 0.03 | 0.973 | .39 |

^a^Exp (β), or the exponential value of β (e^β^), provides the incidence risk ratio for the count model, that is, the association between baseline maternal age and the incidence rate of participants responding to a positive number of text message timepoints.

^b^Exp (β) provides the odds ratio in the zero model, that is, the association between baseline maternal age and the odds of responding to at least 1 text message timepoint.

^c^Adjusted hurdle regression models include maternal age and iself-reported race and ethnicity, income level, and education level.

**Table S9.** Associations between baseline mental health brief measure scores and participants’ number of SMS text message timepoints completed.

| **Continuous Variables** | n | β Count | SE β Count | Exp (β) Count ^b^ | *P* value Count ^d^ | β Zero | SE β Zero | Exp (β) Zero ^c^ | *P* value Zero ^d^ |
| --- | --- | --- | --- | --- | --- | --- | --- | --- | --- |
| Unadjusted | | | | | | | | | |
| Whooley – Baseline | 1001 | -0.032 | 0.011 | 0.969 | *.004* | -0.13 | 0.13 | 0.874 | .3 |
| GAD-2 – Baseline | 1001 | -0.011 | 0.006 | 0.989 | .06 | 0.18 | 0.08 | 1.197 | *.03* |
| Adjusted ^a^ | | | | | | | | | |
| Whooley – Baseline | 896 | -0.023 | 0.012 | 0.977 | *.045* | -0.09 | 0.15 | 0.914 | .56 |
| GAD-2 – Baseline | 898 | -0.008 | 0.006 | 0.992 | .23 | 0.26 | 0.09 | 1.294 | *.007* |

^a^ Adjusted hurdle regression models (with continuous variables) include self-reported race and ethnicity, income level, and education level as covariates.

^b^ Exp (β), or the exponential value of β (e^β^), provides the incidence risk ratio for the count model, that is, the association between baseline mental health measure scores and the incidence rate of participants responding to a positive number of SMS text message timepoints.

^c^ Exp (β) provides the odds ratio in the zero model, that is, the association between baseline mental health measure scores and the odds of responding to at least 1 SMS text message timepoint.

^d^ Italicized values indicate statistical significance.

**Table S10.** Added predictive value of SMS text message response rate on the prediction of postpartum depression and anxiety symptoms by multiple linear regression analyses.

| Outcome | Predictor | n | Response rate Beta | Response rate SE | Response rate R2-change | Response rate *P* value |
| --- | --- | --- | --- | --- | --- | --- |
| Unadjusted | | | | | | |
| EPDS – Postpartum | Whooley – Baseline | 787 | 0.01 | 0.78 | <0.001 | .99 |
| EPDS – Postpartum | EPDS – Baseline | 787 | 0.8 | 0.73 | 0.001 | .27 |
| STAI-S – Postpartum | GAD-2 – Baseline | 771 | -0.08 | 1.79 | <0.001 | .97 |
| STAI-S – Postpartum | STAI-S – Baseline | 767 | 1.41 | 1.73 | 0.001 | .41 |
| Adjusted ^a^ | | | | | | |
| EPDS – Postpartum | Whooley – Baseline | 723 | 0.02 | 0.9 | <0.001 | .98 |
| EPDS – Postpartum | EPDS – Baseline | 725 | 0.87 | 0.84 | 0.001 | .3 |
| STAI-S – Postpartum | GAD-2 – Baseline | 711 | -0.26 | 2.05 | <0.001 | .9 |
| STAI-S – Postpartum | STAI-S – Baseline | 710 | 0.79 | 1.96 | <0.001 | .69 |

^a^ Adjusted regression models include self-reported race and ethnicity, income level, and education level as covariates.
